# Macrophage PTP1B regulates mitochondrial dynamics via the JAK2/STAT3-OPA1 axis and activates the cGAS/STING signaling pathway

**DOI:** 10.3389/fimmu.2025.1644289

**Published:** 2025-10-08

**Authors:** Xuefeng Lei, Diandian Qian, Wenzheng Zhang, Bin’an Zhao, Yabin Li, Hua Hao, Jing Yuan, Le Zhao, Centao Liu

**Affiliations:** ^1^ Department of Orthopaedics, Yangpu Hospital, School of Medicine, Tongji University, Shanghai, China; ^2^ Department of Geriatrics, Zhongshan Hospital, Fudan University, Shanghai, China; ^3^ Department of Sports Medicine, Tongji Hospital, School of Medicine, Tongji University, Shanghai, China; ^4^ School of Life Sciences and Technology, Tongji University, Shanghai, China; ^5^ College of Traditional Chinese Medicine, Inner Mongolia Medical University, Hohhot, Inner Mongolia, China; ^6^ Innovation Center, Clinical Medicine Innovation Park, Shanghai Tenth People’s Hospital Affiliated to Tongji University, Shanghai, China; ^7^ Department of Orthopedics, The Affiliated Changshu Hospital of Nantong University, Changshu No. 2 People’s Hospital, Changshu, Jiangsu, China

**Keywords:** macrophages, PTP1B, tendinopathy, mitochondrial dynamics, JAK2/STAT3 signaling pathway

## Abstract

Tendinopathy is characterized by degenerative changes in tendon tissue, with its pathogenesis closely associated with macrophage-mediated chronic inflammation and mitochondrial dysfunction. Bioinformatics analysis of tendinopathic tissues revealed a significant upregulation of protein tyrosine phosphatase 1B (PTP1B) in macrophages, which accompanied with robust immune activation and marked Janus Kinase 2/Signal Transducer and Activator of Transcription 3 (JAK2/STAT3) signaling pathway inhibition. In tendinopathy mouse models, both pro-inflammatory cytokines and PTP1B were found to be highly expressed in tendon tissues. However, conditional deletion of *Ptpn1* (encoding PTP1B, Ptpn1^-/-^) in macrophages significantly alleviated tendon inflammation and fibrosis, indicating a strong association between PTP1B and tendinopathy. Mechanistically, in vivo experiments demonstrated that macrophage PTP1B suppressed STAT3 activation by inhibiting JAK2 phosphorylation, and inhibited the mitochondrial fusion protein Optic Atrophy1 (OPA1), resulting in mitochondrial fragmentation and mitochondrial DNA (mtDNA) release. This process activated the Cyclic GMP-AMP synthase/Stimulator of interferon genes (cGAS/STING) pathway, elevating the levels of inflammation and exacerbating tendon injury. In summary, macrophage PTP1B was shown to regulate mitochondrial dynamics via the JAK2/STAT3-OPA1 axis and trigger inflammation through activation of the cGAS/STING pathway, representing a key mechanism underlying the progression of tendinopathy. Targeting PTP1B or associated pathways may provide novel therapeutic strategies for tendinopathy.

## Introduction

Tendinopathy is a degenerative disease of tendon tissue characterized by chronic inflammation and functional impairment (1), clinically manifesting as stiffness, pain, and restricted mobility, thereby severely affecting patients’ quality of life. It predominantly affects athletes, manual laborers, and the elderly, with common sites including the rotator cuff, Achilles tendon, and patellar tendon (2). The prevalence and incidence of lower limb tendinopathy, as indicated by the epidemiological evidence, are 11.83 and 10.52 cases per 1000 person-years, respectively, and increase with age (3). Conventional treatments, such as nonsteroidal anti-inflammatory drugs and surgery, can provide symptomatic relief but are limited in efficacy and prone to relapse. In some cases, patients may progress to tendon rupture or chronic fibrosis (4). Therefore, elucidating the mechanisms of tendinopathy for therapeutic targets remains a critical challenge in clinical management.

Recent studies have indicated that tendinopathy involves an imbalance between inflammation and tissue repair, with macrophages playing a central role (5–7). In the early stage of injury, mechanical stress or microtrauma induces an immune reaction, leading to the M1 macrophage recruitment in the injury site and intensifying inflammatory levels (8). Although this inflammatory response is essential for tissue repair, excessive macrophage activation promotes the secretion of matrix metalloproteinases (MMPs), resulting in collagen disorganization and tendon structural damage (9). During disease progression, macrophages fail to polarize toward the anti-inflammatory M2 phenotype, thus impairing tissue repair and promoting fibrosis (10). Moreover, disruption of mitochondrial dynamics (i.e., fusion-fission balance) has been identified as a key driver of tendinopathy, contributing to reactive oxygen species (ROS) accumulation, energy metabolism disorders, and mitochondrial DNA (mtDNA) leakage, further activating immune responses and forming a vicious cycle (11, 12). However, the mechanisms linking macrophage polarization and mitochondrial dynamics in the context of tendinopathy remain poorly understood.

Protein tyrosine phosphatase 1B (PTP1B), a crucial regulator of cellular signaling pathways, involves in inflammation, metabolism, and tissue homeostasis. It has been implicated in various metabolic and chronic inflammatory diseases (13, 14). In rheumatoid arthritis, PTP1B inhibition was found to alleviate synovial inflammation by suppressing the levels of NF-κB signaling (15). Similarly, in cardiac fibrosis, PTP1B deficiency attenuated collagen deposition by modulating TGF-β/Smad signaling (16). However, the role of PTP1B in tendinopathy has rarely been explored.

**Figure 1:**
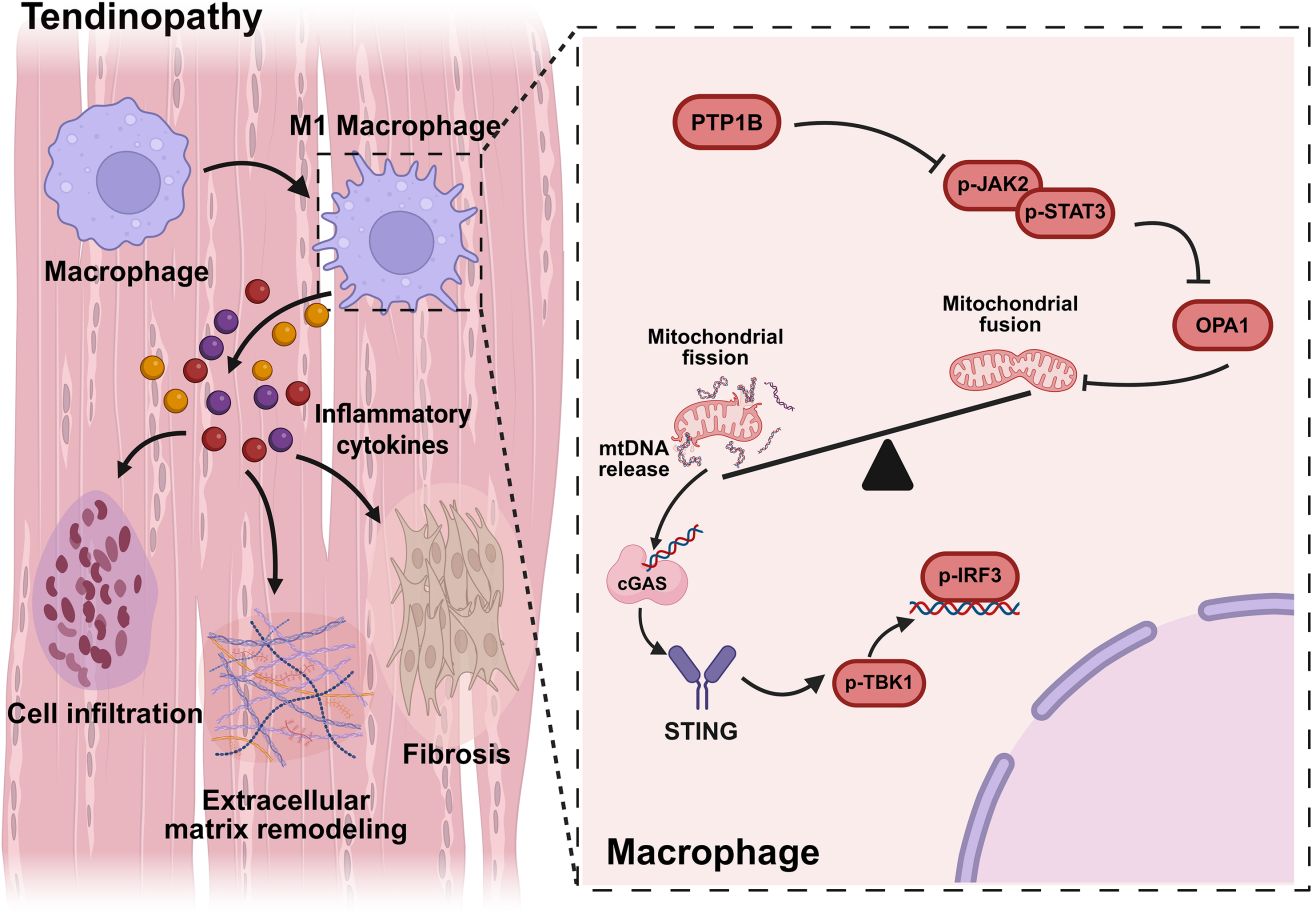
In diseased tendons, macrophage PTP1B inhibits the JAK2/STAT3 signaling pathway, resulting in reduced the expression of OPA1 and the leakage of mtDNA, which activates the cGAS/STING signaling and exacerbates tendinopathy.

Bioinformatics analysis was first performed using bulk RNA sequencing (RNA-seq) data (Gene Expression Omnibus, GSE26051) and single-cell RNA sequencing (scRNA-seq) data of tendinopathy (National Genomics Data Center, HRA002325). An increased proportion of M1 macrophages and significant upregulation of PTP1B in macrophages were observed, along with marked Janus Kinase 2/Signal Transducer and Activator of Transcription 3 (JAK2/STAT3) signaling inhibition. In the collagenase-induced murine model of tendinopathy, the elevated expression of macrophage PTP1B and pro-inflammatory cytokines was also identified. Notably, PTP1B deletion significantly mitigated tendon inflammation and fibrosis. As shown in **Figure 1**, mechanistic studies revealed that PTP1B suppressed the JAK2/STAT3 signaling cascade in macrophages, leading to decreased expression of Optic Atrophy1 (OPA1), a protein essential for mitochondrial fusion. This resulted in increased mitochondrial fragmentation and mtDNA leakage, which activated the Cyclic GMP-AMP synthase/Stimulator of interferon genes (cGAS/STING) signaling in sequence, further aggravating inflammation and tissue damage. In short, this study reveals that macrophage PTP1B regulates mitochondrial dynamics and immune responses in tendinopathy, suggesting a promising target in tendinopathy therapy.

## Materials and methods

### Acquisition and analysis of public database data

Data were obtained from the public database GSE26051, including sample identifiers from the control group (GSM639748–GSM639770) and the tendinopathy group (GSM639771–GSM639793). All these samples were collected from 23 patients with tendinopathy, and a non-lesional tendon and a lesional tendon (~3 mm³) were collected from each patient respectively. The non-lesional tendons were used as the control group, and the lesional tendons were used as the tendinopathy group. Gene expression data were obtained through Affymetrix Human Genome U133 Plus 2.0 Array platform. The scRNA-seq data were obtained from National Genomics Data Center (HRA002325), and were generated using the Illumina NovaSeq 6000 platform.

For bulk RNA-seq, the CIBERSORT algorithm was used to quantify the proportion of immune cell subsets to determine the abundance changes of the cells. Meanwhile, the correlation network among immune cell subsets was calculated through the CIBERSORT results to reveal the synergistic or antagonistic relationship.

For scRNA-seq, after screening out CD45^+^ immune cells, 10 immune cell subpopulations were generated by unsupervised clustering, including macrophages, monocytes, and others. Differentially expressed analysis was conducted (|log2FC| > 1, p.adj < 0.05). Enriched signaling pathways were identified using gene set enrichment analysis (GSEA). Focusing on macrophage subpopulations, expression changes of key genes were analyzed to assess JAK2/STAT3 signaling activity.

### Animals

Conditional *Ptpn1* knockout mice in macrophages (*LysM^Cre^Ptpn1^flox/flox^*) were generated using CRISPR-Cas9 technology (Cyagen Biosciences Inc). Male C57BL/6 mice (8 weeks) were acquired from Shanghai Model Organisms Center, Inc. A volume of 20 μL collagenase I solution (15 mg/mL) was injected around the Achilles tendon in mice for 3 weeks; control mice received solvent injections. H-151 (a STING inhibitor) was injected intraperitoneally (10 mg/kg) once every 2 days for 3 times in 1 week. After completing the experimental period, the mice were euthanized, and tendon tissues were harvested. The tissues were prepared as paraffin sections for histological analysis through fixation, dehydration, and clearing procedures.

### Immunoblotting

The total protein was extracted and quantified. After conducting the steps related to immunoblotting, the bands were visualized via the iBright (Thermo). The quantification of the band intensities performed via ImageJ. The primary antibody for immunoblotting was PTP1B Rabbit mAb (A23035, Abclonal).

### Histological staining

Hematoxylin and eosin (H&E) staining and Masson staining was conducted on the tendon sections.

After deparaffinization, rehydration, and staining, sections were examined microscopically to assess inflammatory infiltration, tissue damage, and fibrosis. For 3,3′-diaminobenzidine (DAB) staining procedure, tendon sections were subjected to an incubation with anti-PTP1B (Abclonal, A23035), COL1A1 (CST, 72026), and COLIII (Abcam, ab278080) antibodies, followed by S-vision poly-HRP conjugated goat anti-rabbit IgG (H+L) secondary antibody. Hematoxylin counterstaining was applied before sealing the slides. For immunofluorescence staining, the overnight incubations with primary antibodies (F4/80/PTP1B, F4/80/Arg1, F4/80/p-JAK2, F4/80/p-STAT3, F4/80/p-STING, F4/80/p-TBK1, F4/80/p-IRF3, F4/80/OPA1, F4/80/DRP1, F4/80/iNOS). were conducted on the mouse tendon sections. Appropriate secondary antibodies were applied (Alexa Fluor 488 (ab150077) and 594 (ab150080), Cy3(A0516, Beyotime) for 1 hour, followed by DAPI nuclear staining. All images were acquired using an Olympus DP74 imaging system, and relative protein expression was quantified using ImageJ. The following antibodies were used: F4/80 Rabbit mAb (30325), Phospho-IRF3 Rabbit mAb (29047), Phospho-TBK1/NAK Rabbit mAb (5483), Phospho-STING Rabbit mAb (62912, CST), Arginase-1 Rabbit mAb (A25808), OPA1 Rabbit pAb (A9833), DRP1 Rabbit mAb (A21968, Abclonal), Anti-PTP1B (ab245984), Anti-iNOS (ab178945), Anti-JAK2 (phospho Y1007 + Y1008) (ab32101), and Anti-STAT3 (phospho Y705) (ab76315, Abcam).

### Histopathological scoring

Histopathological staining was scored based on three parameters: injury, inflammation, and repair. Injury scoring: 0, no evident injury; tendon structure normal with well-aligned cells; 1, mild injury with minor collagen fiber rupture or damage and minimal cellular infiltration; 2, moderate injury with apparent tendon rupture, more extensive collagen fiber damage, and noticeable inflammatory cell infiltration; 3, severe injury with widespread tendon rupture or destruction, complete loss of collagen fibers, and significant inflammatory response with cellular infiltration. Inflammation scoring: 0, inflammatory cell infiltration free; 1, mild inflammation and cellular infiltration; 2, moderate inflammatory response with an evident increase in inflammatory cells; 3, severe inflammation with extensive inflammatory cell infiltration and tissue necrosis. Repair scoring: 0, normal tendon structure with intact collagen fibers; 1, mild repair with irregular collagen fiber alignment; 2, partial repair with proliferated collagen fibers but poor alignment; 3, complete or abnormal reconstruction with significantly rebuilt collagen fibers, possibly irregular or fibrotic.

### Biomechanical analysis

The complex, including the calcaneus-tendon-muscle belly was completely isolated and placed in pre-cooled PBS at 4°C The surrounding fat and fascia tissue were finely removed to obtain the tendon tissue. The cross-sectional area was continuously measured at three points (± 0.01mm) in the middle of the tendon using a caliper (Mitutoyo1), and the average value was taken as the calculation reference value. The dual-column mechanical testing system (Instron 5848 MicroTester) is equipped with a 500N high-precision load sensor. After the tendon sample was installed, a preload of 0.1N was applied for 60 seconds, and then the sample was stretched at a quasi-static rate of 0.3mm/s until it broke. The ultimate load (N) is read through the load-displacement curve. The ultimate stress (N/mm ²) is calculated as the ultimate load divided by the cross-sectional area. Young’s modulus (N/mm ²) is calculated by the linear slope of the stress-displacement curve. During the testing process, the sample was kept moist with 0.9% salt water.

### Quantitative real-time PCR

After the extract of total RNA via TRIzol, cDNA was synthesized following the manufacturer (RR064A, Takara). Real-time PCR was conducted using PrimePCRTM (Bio-Rad). Reverse transcription conditions: 42°C for 5min, 95°C for 5 s, 1 cycle. PCR conditions: 95°C for 5 s, 60°C for 20 s, 40 cycles. Primers used are listed in **Table 1**.

**Table 1:** Primers.

Gene name	Primer sequence (5’→3’)
*Ptpn1-F*	GACCTGGTGAAGACCATCCA
*Ptpn1-R*	TGGTGAAGGTGCTGTTGAAG
*Tnf-α-F*	TACTGAACTTCGGGGTGATTGGTCC
*Tnf-α-R*	CAGCCTTGTCCCTTGAAGAGAAC
*Il-6-F*	CAGCCTTGTCCCTTGAAGAGAAC
*Il-6-R*	GGAAATTGGGGTAGGAAGGA
*Il-1β-F*	GCACTACAGGCTCCGAGATGAAC
*Il-1β-R*	TTGTCGTTGCTTGGTTCTCCTTGT
*Cox1-F*	CCGACAGCCTAGCCATAGAC
*Cox1-R*	GGTGGTTGGTGTAAAGGAAG
*Ifn-β-F*	CAGCTCCAAGAAAGGACGAAC
*Ifn-β-R*	GGCAGTGTAACTCTTCTGCAT
*Nd1-F*	ATGGCCTTCCTCACCCTAGT
*Nd1-R*	GTTAGGGGGCGTATGGGTTC
*Gapdh-F*	AGGTCGGTGTGAACGGATTTG
*Gapdh-R*	TGTAGACCATGTAGTTGAGGTCA

### Flow cytometry

To assess macrophage polarization within tendon tissues, the cell suspensions were stained with the specific antibodies for M1 type (CD86) and M2 type (CD206) macrophage markers. The proportions of M1 and M2 macrophages were quantified via flow cytometer (BD FACSCelesta). FITC Fluorescent Anti-F4/80 antibody (ab105155), Alexa Fluor^®^ 594 fluorescent Anti-CD86 antibody (ab303578), Alexa Fluor^®^ 594 fluorescent Anti-Mannose Receptor antibody (ab318108) and anti-PTP1B antibody (ab201974) were provided by Abcam.

### Enzyme linked immunosorbent assay

After preparing the suspensions via homogenization to obtain the soluble proteins from tendon, these samples were subjected to ELISA (Cloud-Clone Corp) to determine levels of several cytokines.

### Statistical analysis

All data were presented as mean ± standard deviation (M ± SD), Student’s t-test and One-way ANOVA with Tukey’s *post hoc* test were carried out to determine the differences between several groups via GraphPad Prism 9.0. *p < 0.05, **p < 0.01, ***p < 0.001 and ****p < 0.0001 were considered statistically significant.

## Results

### Bioinformatic analysis of tendinopathy

**Figure 2:**
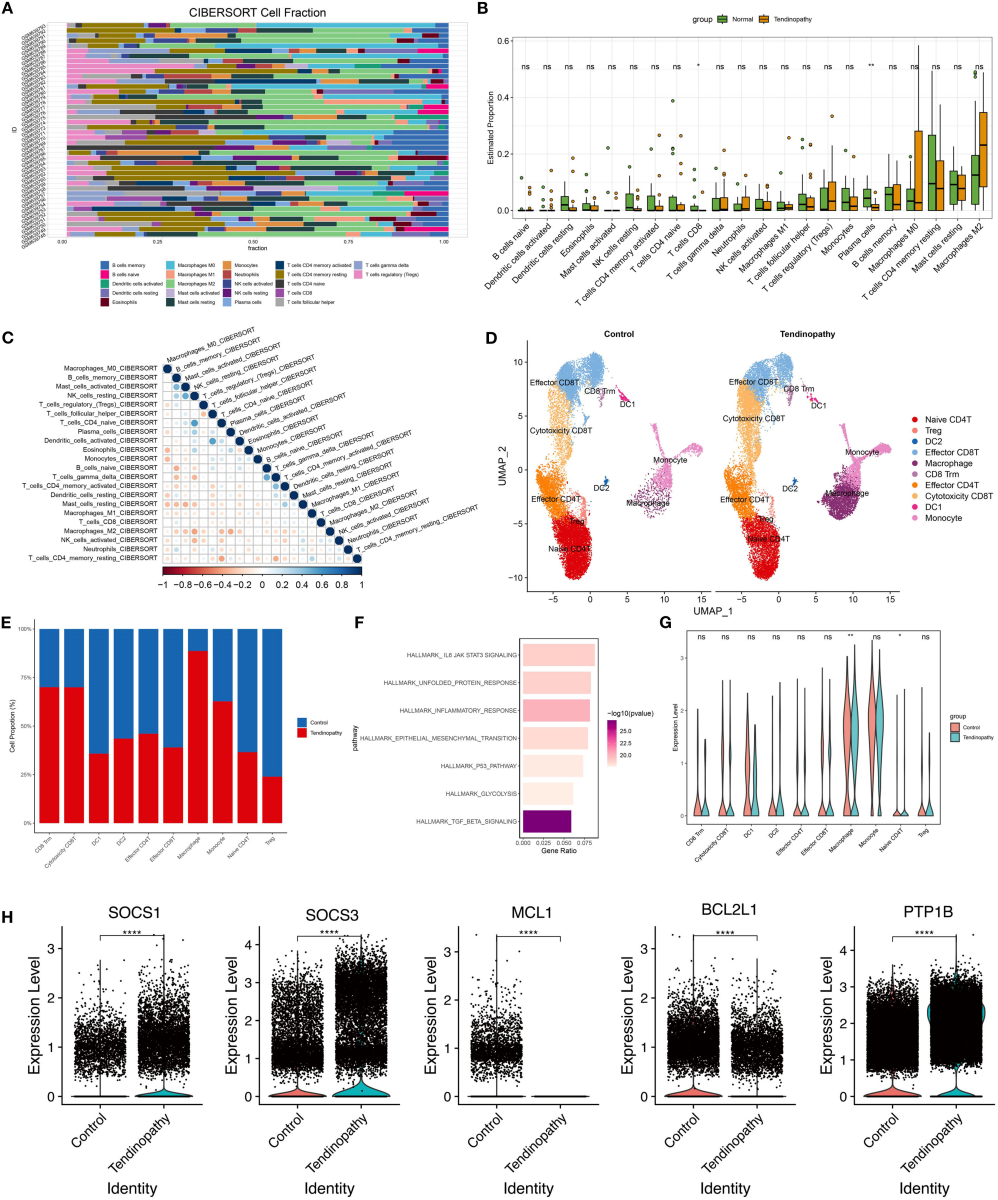
Bioinformatics analysis of tendinopathy. **(A)** Cell types, **(B)** cell proportions, and **(C)** immune cell interactions in GSE26051 were analyzed using the CIBERSORT tool; **(D)** UMAP projection of all immune cell clusters and **(E)** relative proportions of each immune cell population in control and tendinopathy group; **(F)** Significantly enriched pathways by GSEA analysis; **(G)** Expression levels of JAK/STAT3 genes in control and tendinopathy; **(H)** Expression levels of SOCS1, SOCS3, BCL2L1, MCL1, and PTP1B in macrophages from control and tendinopathy. DC1, conventional dendritic cell type 1; DC2, conventional dendritic cell type 2.

Gene expression data related to tendinopathy were obtained from the public database (GSE26051). Bioinformatic analysis using the CIBERSORT tool was performed on normal control samples (GSM639748-GSM639770) and tendinopathy samples (GSM639771-GSM639793). The results indicated augmented proportions of M1 macrophages and CD4^+^ memory activated T cells in the tendinopathy group, with no significant changes observed in B cells and NK cells (**Figure 2A**). Further analysis revealed significantly decreased proportions of CD8^+^ T cells (P < 0.05) and plasma cells (P < 0.01) in the tendinopathy group (**Figure 2B**), suggesting that cytotoxic T cell-mediated immune response may not be the primary pathological mechanism in tendinopathy.

CIBERSORT algorithm analysis of immune cell subtypes indicated a collaborative or synergistic relationship between M1 macrophages and T cell activation (**Figure 2C**), implying a synergistic pro-inflammatory interaction contributing to tissue damage and chronic inflammation. Additionally, regulatory T (Treg) cells were positively correlated with M2 macrophages, suggesting potential cooperation in limiting excessive inflammation, although their low proportions may render them insufficient to counteract inflammation (**Figure 2C**). These findings suggest that macrophages constitute a key immune cell population in tendinopathy.

To further investigate immune cell roles in tendinopathy, CD45^+^ immune cells were screened based on the scRNA-seq data of tendinopathy (HRA002325). Unsupervised clustering generated ten immune cell clusters representing robust cell types in both normal and diseased tendons (**Figure 2D**). Although the basic cell types remained similar, immune cell composition differed significantly between groups. Quantitative analysis of cluster percentages revealed a notable increase in macrophages, monocytes, CD8^+^ Tissue-resident Memory T cells (CD8 Trm), and cytotoxic CD8 T cells in tendinopathy (**Figures 2D, E**). Transcriptomic differences among clusters were analyzed (|log2FC|> 1 and p.adj < 0.05) to identify the genes which were undergo significant alterations in tendinopathy. GSEA indicated enrichment of TGF-β signaling, inflammatory response, and JAK/STAT3 signaling pathway in diseased tendons (**Figure 2F**). Notably, JAK/STAT3 signaling was most downregulated significantly in macrophages (**Figure 2G**). Moreover, SOCS1 and SOCS3 were upregulated, and BCL2L1 and MCL1 were downregulated in the macrophages of Tendinopathy (**Figure 2H**). Among them, SOCS1 and SOCS3 are known negative regulators of the JAK/STAT pathway. Their upregulation indicated significant inhibition of JAK/STAT signaling pathway (17–19). Additionally, Ptpn1 (PTP1B), a key regulator of JAK2/STAT3 signaling, was significantly upregulated in tendinopathy macrophages (**Figure 2H**). This suggests a multi-layered suppression mechanism of JAK2/STAT3 signaling in tendinopathy. However, the expression level of PTP1B showed no significant differences among the other 9 immune cell clusters (**Supplementary Figure S1**). Prior studies demonstrated that PTP1B hydrolyzes phosphorylated JAK2 to modulate STAT3 activity, contributing to astrocyte dysfunction and hypothalamic inflammation (20, 21). These findings suggest a potential association between elevated PTP1B expression and suppressed JAK2/STAT3 signaling in macrophages, potentially exacerbating inflammation and tissue damage in tendinopathy.

### Establishment of the collagenase-induced mouse tendinopathy model and the potential pathological role of PTP1B

**Figure 3:**
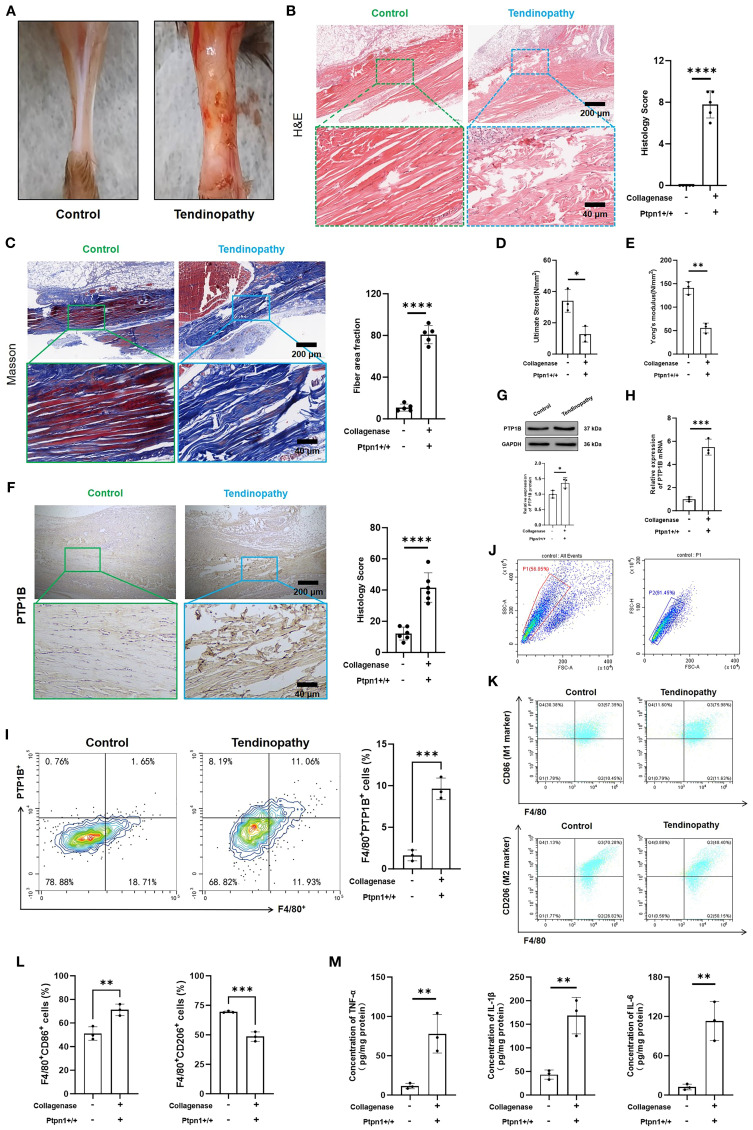
Establishment of a collagenase-induced mouse tendinopathy model and the potential pathological role of PTP1B. **(A)** Images of mouse tendons; **(B)** H&E staining and histological scores of mouse tendons (scale bar, 200 μm, 40 μm, n = 5); **(C)** Masson staining and fiber area fraction statistics (scale bar, 200 μm, 40 μm, n = 5); **(D)** Ultimate stress and **(E)** Young’s modulus measurements (n = 3); **(F)** Immunohistochemical (IHC) staining and histological scores of PTP1B in tendon tissues (scale bar, 200 μm, 40 μm, n = 5); **(G)** Immunoblotting and statistical analysis of PTP1B (n = 3); **(H)** Relative mRNA expression levels of PTP1B in tendon tissues (n = 3); **(I)** Quantification of F4/80^+^PTP1B^+^ cells in tendons via flow cytometry. (n = 3); **(J)** Quantification of M1 macrophages (F4/80^+^CD86^+^) and M2 macrophages (F4/80^+^CD206^+^) via flow cytometry (n = 3); **(K)** ELISA detection of TNF-α, IL-6, and IL-1β (n = 3). (*p < 0.05, **p < 0.01, ***p < 0.001, ****p < 0.0001).

A mouse tendinopathy model was established using collagenase to evaluate tendon injury and pathological changes. Contrary to the control group, the tendinopathy model exhibited tendon thickening, darkened coloration, and increased stiffness (**Figure 3A**), indicating significant morphological changes and tendon injury. H&E and Masson staining of tendon sections were performed. In healthy controls, tendon fibers were well-organized with evenly distributed vasculature and absence of inflammatory infiltration. In contrast, tendinopathic tendons displayed severe injuries, including ruptured muscle fibers, disrupted architecture, and substantial inflammatory infiltration (**Figure 3B**). Masson staining confirmed collagen fiber disorganization and increased fibrosis in the tendinopathy group (**Figure 3C**), Mechanical properties were also affected; ultimate stress (**Figure 3D**) and Young’s modulus (**Figure 3E**) were reduced markedly in the tendinopathy group, suggesting a compromised biomechanical performance of tendinopathic tendons.

Consistent with bioinformatics analysis, the PTP1B level was also elevated in pathological tendon tissues, accompanied by a marked increase in histological scores (**Figure 3F**), suggesting a pivotal role of PTP1B in tendinopathy. Concurrently, both immunoblotting (**Figure 3G**) and qRT-PCR (**Figure 3H**) proved the upregulation of PTP1B in diseased tendon tissues. However, there was no significant change in the expression level of PTP1B in skeletal muscle and synovial tissue (**Supplementary Figure S2**). Furthermore, the expression of macrophage-derived PTP1B within tendon tissues was examined, with macrophages identified by F4/80 staining. These findings above proved that the level of macrophage PTP1B in was elevated markedly in diseased tendons (**Figure 3I**; **Supplementary Figure S3**), which may influence macrophage polarization. As expected, the proportion of M1 macrophages (F4/80^+^CD86^+^) was increased, while the proportion of M2 macrophages (F4/80^+^CD206^+^) was reduced in pathological tendon tissues (**Figure 3J**), indicating that elevated PTP1B expression may promote M1 polarization and enhance immune and inflammatory responses. This was supported by ELISA, which revealed the augmented secretion of pro-inflammatory cytokines (TNF-α, IL-6, IL-1β) in diseased tendons than the normal tendons (**Figure 3K**). The augmented secretion of these cytokines was consistent with histopathological observations of inflammatory cell infiltration and tissue damage.

### Conditional knockout of macrophage Ptpn1 alleviates tendon injury and inflammation

**Figure 4:**
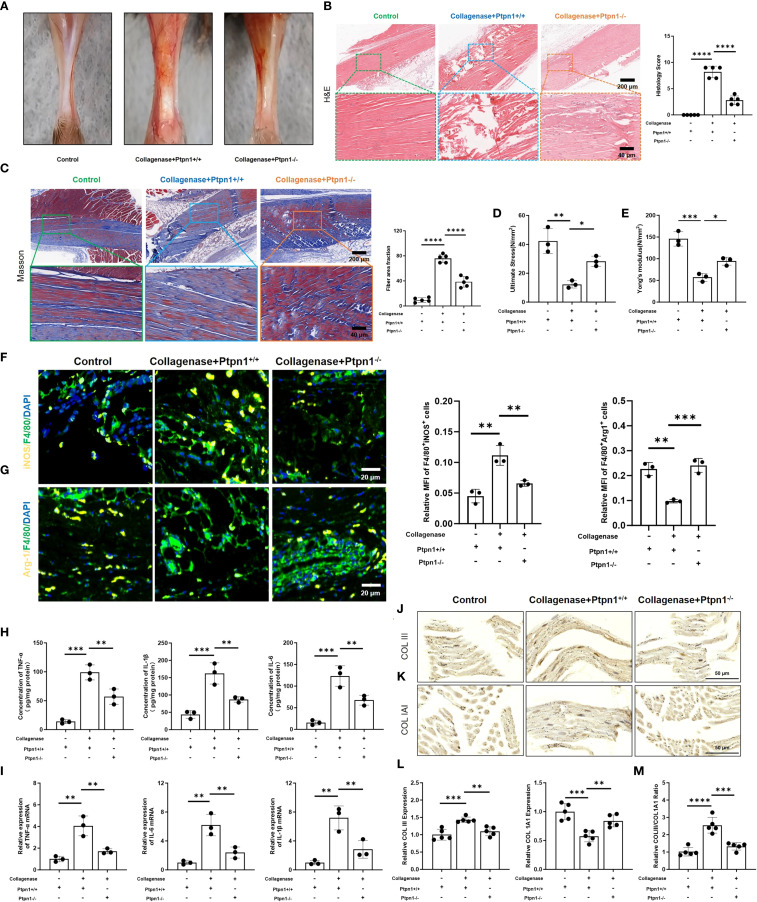
Conditional deletion of Ptpn1 in macrophages attenuates tendon injury and inflammation. **(A)** Images of mouse tendons; **(B)** H&E staining and histological scores of mouse tendons (scale bar, 200 μm, 40 μm, n = 5); **(C)** Masson staining and fiber area fraction statistics (scale bar, 200 μm, 40 μm, n = 5); **(D)** Ultimate stress and **(E)** Young’s modulus measurements (n = 3); **(F)** Immunofluorescence staining and statistical analysis of M1 macrophages (F4/80^+^iNOS^+^) and **(G)** M2 macrophages (F4/80^+^Arg1^+^) in tendon tissues (scale bar, 100 μm, n = 3); **(H)** ELISA detection of TNF-α, IL-6, and IL-1β (n = 3); **(I)** Relative mRNA expression levels of TNF-α, IL-6, and IL-1β in tendon tissues (n = 3); **(J)** IHC staining of COLIII and **(K)** COL1A1 in tendon tissues (scale bar, 50 μm, n = 3); **(L)** Statistical analysis of the relative expression levels of COLIII and COL1A1; **(M)** Statistical analysis of the ratio of COLIII to COL1A1 expression levels. (*p < 0.05, **p < 0.01, ***p < 0.001, ****p < 0.0001).

To investigate the function of PTP1B in tendinopathy, we generated macrophage-specific Ptpn1 (Ptpn1^-/-^) knockout mice via CRISPR-Cas9 system. Mice were then assigned to three groups: control, collagenase + Ptpn1^+/+^ (normal Ptpn1 expression with collagenase treatment), and collagenase + Ptpn1^-/-^ (macrophage-specific Ptpn1 knockout with collagenase treatment). Tendons in the control group displayed normal morphology without evident injury, while those in the collagenase + Ptpn1^+/+^ group exhibited severe damage. Notably, tendon damage in the collagenase + Ptpn1^-/-^ group was alleviated to a certain extent (**Figure 4A**). Histopathological evaluation using H&E staining (**Figure 4B**) and Masson staining (**Figure 4C**) further confirmed that macrophage-specific Ptpn1 knockout mitigated collagenase-induced tendon injury and fibrosis. Similarly, the mechanical properties of tendon tissues, including ultimate stress and Young’s modulus, which were adversely affected by collagenase, were partially restored in the Ptpn1 knockout group (**Figures 4D, E**).

Immunofluorescence staining was used to investigate the association between PTP1B and M1 macrophage polarization. As anticipated, fluorescence intensity of M1 macrophages (F4/80^+^iNOS^+^) was reduced upon Ptpn1 knockout (**Figure 4F**), whereas fluorescence intensity of M2 macrophages (F4/80^+^Arg1^+^) was elevated (**Figure 4G**), indicating that PTP1B promotes M1 polarization. Consequently, the inflammatory cytokines (TNF-α, IL-6, IL-1β) levels were decreased, and this effect was attenuated in the absence of Ptpn1 (**Figures 4H, I**). type I collagen (COL1A1) and type III collagen (COLIII) are critical constituents of tendon extracellular matrix (ECM) (22, 23), critical for structural integrity and function. IHC staining of tendon tissues showed that both COL1A1 and COLIII were evenly distributed in the control group. Following collagenase treatment, COL1A1 expression was reduced, leading to an increased COLIII/COL1A1 ratio (**Figures 4J-M**). However, in collagenase-treated Ptpn1^-/-^ mice, COL1A1 levels were restored, and the COLIII/COL1A1 ratio was reduced (**Figures 4J-M**). Overall, macrophage-derived PTP1B in pathological tendons was found to promote M1 polarization, inflammation, and fibrosis, while Ptpn1 knockout attenuated these deleterious effects, supporting its potential as a therapeutic target in tendinopathy.

### PTP1B regulates mitochondrial dynamics and cGAS/STING pathway via the JAK2/STAT3–OPA1 axis

**Figure 5:**
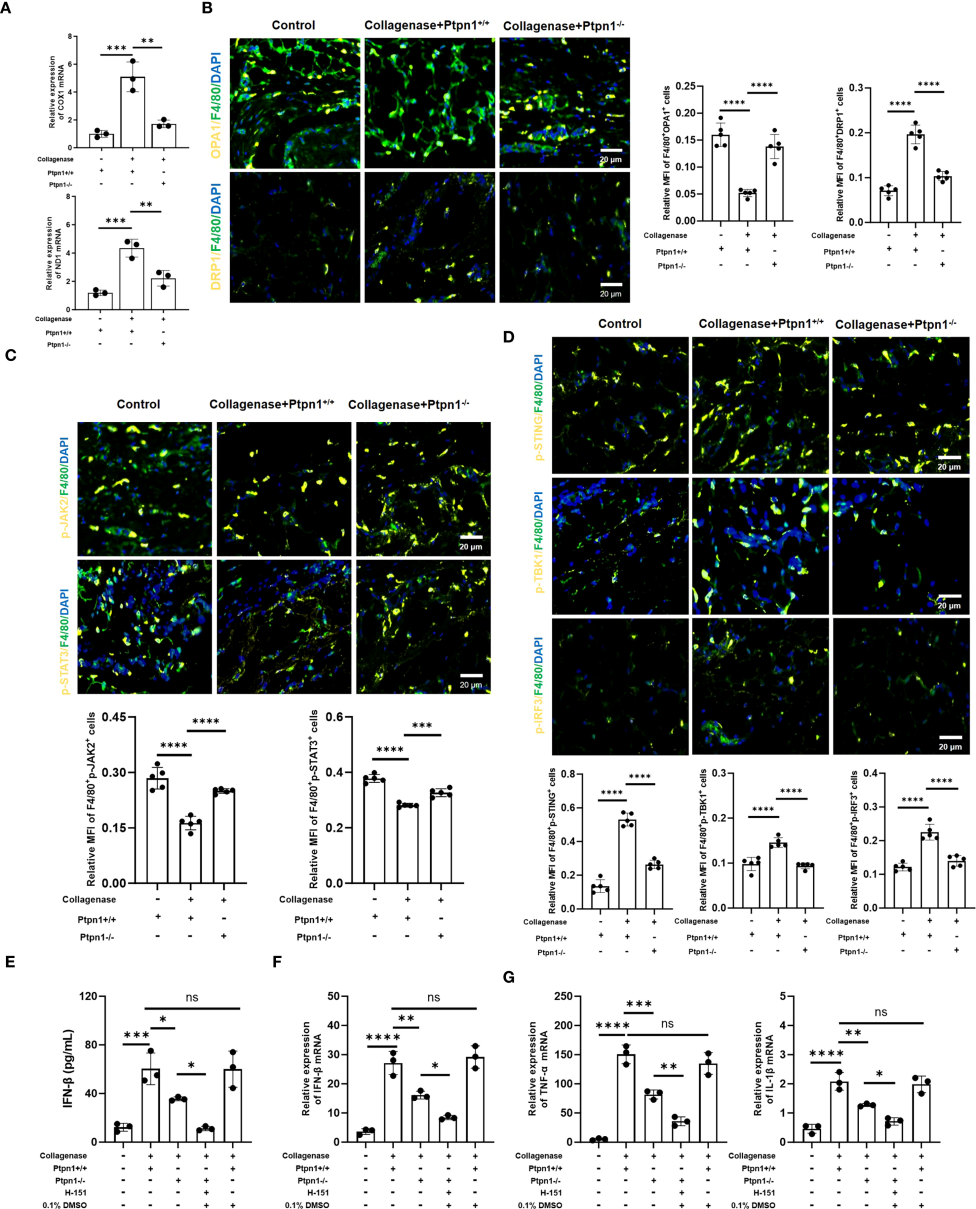
PTP1B regulates mitochondrial dynamics and the cGAS/STING signaling pathway via JAK2/STAT3-OPA1 axis. **(A)** The mRNA Expression level of COX1 and ND1 determined by qRT-PCR; **(B)** Immunofluorescence staining of OPA1 (F4/80^+^OPA1^+^) and DRP1 (F4/80^+^DRP1^+^) in macrophages from tendon tissues and corresponding statistical analysis (scale bar, 50 μm, n = 5); **(C)** Expression levels and statistical analysis of p-JAK2 (F4/80^+^p-JAK2^+^) and p-STAT3 (F4/80^+^p-STAT3^+^) (scale bar, 50 μm, n = 5); **(D)** Expression levels and statistical analysis of p-STING (F4/80^+^p-STING^+^), p-IRF3 (F4/80^+^p-IRF3^+^), and p-TBK1 (F4/80^+^p-TBK1^+^) (scale bar, 50 μm, 100 μm, n = 5). **(E)** ELISA detection of IFN-β in tendons (n = 3). **(F)** The mRNA expression of f IFN-β in tendons (n = 3). The mRNA expression of TNF-α and IL-1β in tendons (n = 3) (*p < 0.05, **p < 0.01, ***p < 0.001, ***p < 0.0001).

Cell function and homeostasis are regulated not only by inflammatory cytokines but also by various intracellular organelles. As the center of energy metabolism, mitochondria exert profound effects on cellular function when altered. Previous studies have shown that inflammatory macrophages experience mitochondrial damage that leads to mtDNA leakage and activation of the cGAS/STING pathway (24). Thus, the cytosolic mtDNA level was quantified and the mRNA expression of COX1 and ND1 was elevated in the collagenase + Ptpn1^+/+^ group compared to controls, indicating increased mtDNA content, while this effect was suppressed in the absence of Ptpn1 (**Figure 5A**). mtDNA leakage has been associated with impaired mitochondrial fusion (25, 26). Therefore, the expression of OPA1, a regulator of mitochondrial fusion and membrane integrity (27), was assessed. The expression level of OPA1 (F4/80^+^OPA1^+^) was significantly reduced in the collagenase + Ptpn1^+/+^ group (**Figure 5B**), whereas Ptpn1 deletion restored its expression, indicating that macrophage-derived PTP1B induces fusion impairment. Increased expression of Dynamin-related protein 1 (DRP1), a mitochondrial fission-related protein, in the same group further supported this finding (**Figure 5B**). Existing studies have shown that STAT3 can directly bind to the promoter of OPA1 (28, 29). Combined with our bioinformatics analysis, it was identified that JAK2/STAT3 acts downstream of PTP1B, and activated STAT3 promotes OPA1 expression (28, 30). Therefore, the hypothesis was formulated that the impairment of mitochondrial fusion by macrophages is due to the downregulation of OPA1, which is a consequence of the inhibition of the JAK2/STAT3 signaling by PTP1B. As expected, collagenase treatment suppressed the phosphorylation levels of JAK2 and STAT3, whereas Ptpn1 knockout restored their activation (**Figure 5C**), suggesting that PTP1B-mediated inhibition of JAK2/STAT3 signaling leads to fusion defects and mtDNA release. mtDNA leakage was found to activate the cGAS/STING pathway, triggering innate immunity and amplifies inflammation. The expression of the cGAS/STING pathway-related proteins (p-STING, p-IRF3, p-TBK1) were increased in the collagenase + Ptpn1^+/+^ group, indicating pathway activation. This activation was suppressed upon Ptpn1 deletion (**Figure 5D**). In addition, we adding H-151 (a STING inhibitor) to Ptpn1^-/-^ mice via intraperitoneal injection, and the results indicated that the level of IFN-β (a downstream product of the cGAS/STING signaling pathway) was further suppressed (**Figures 5E, F**). Furthermore, the levels of TNF-α and IL-1β were further decreased (**Figure 5G**), indicating that PTP1B is a downstream necessary mediator for the pro-inflammatory effect of cGAS/STING. Collectively, PTP1B in pathological tendon tissues was shown to induce mitochondrial fusion impairment and mtDNA release by inhibiting the JAK2/STAT3–OPA1 axis, thereby activating the cGAS/STING pathway and exacerbating immune and inflammatory responses, ultimately contributing to tendinopathy progression.

## Discussion

This study elucidated the role and mechanisms of macrophage-derived PTP1B in tendinopathy. Integrating bioinformatics and *in vivo* experiments demonstrated that macrophage PTP1B in pathological tendons inhibits the JAK2/STAT3 signaling pathway, downregulates the mitochondrial fusion protein OPA1, induces mitochondrial fusion defects and mtDNA leakage, and activates the cGAS/STING pathway. This cascade culminates in heightened inflammation injury of tendon tissues. In collagenase-induced tendinopathy mouse models, macrophage-specific knockout of Ptpn1 alleviated tendon damage, inflammatory cell infiltration, and fibrosis while suppressing cGAS/STING pathway activation. These findings not only reveal a mechanistic link between macrophage PTP1B-mediated chronic inflammation and mitochondrial dynamics in tendinopathy but also provide a theoretical basis for targeting PTP1B and associated signaling pathways in therapy.

Tendinopathy, characterized by chronic inflammation and tissue degeneration, involves complex pathophysiological mechanisms that remain incompletely understood. This study is the first to associate PTP1B with mitochondrial dynamic imbalance and immune activation in tendinopathy. Previous studies have shown that PTP1B plays a key role in metabolic and inflammatory diseases, yet its involvement in tendinopathy has been scarcely reported. Our bioinformatics analysis and experiments *in vivo* revealed that M1 macrophages were significantly enriched in the lesion tissues of tendinopathy mice, and the expression level of Ptpn1 was specifically elevated in the macrophage subsets, while no significant changes were observed in other immune cells such as T cells and dendritic cells, suggesting that the polarization imbalance of macrophages and the upregulation of PTP1B may be the core driving factors of the continuous inflammation of tendinopathy.

Notably, the JAK2/STAT3 pathway may play dual roles in tissue repair: while STAT3 activation supports proliferation and anti-inflammatory responses, excessive activation may induce fibrosis. This study demonstrated that elevated PTP1B expression in macrophages of pathological tendons suppresses JAK2/STAT3 signaling, thereby impairing mitochondrial fusion and preventing the shift toward the M2 anti-inflammatory phenotype, leading to chronic inflammation. This finding is consistent with prior studies on the role of STAT3 in maintaining mitochondrial dynamics (31), further underscoring the pivotal role of JAK2/STAT3 in preserving cellular homeostasis. Furthermore, we noted elevated expression of SOCS1 and SOCS3 in macrophages from tendinopathic tissues, which are established inhibitors of JAK/STAT signaling. While PTP1B dephosphorylates JAK2, SOCS proteins may further reinforce the inhibition through feedback mechanisms. This synergistic suppression likely exacerbates the impairment of mitochondrial fusion and anti-inflammatory responses. Future investigations should delineate the relative contributions of PTP1B and SOCS proteins to JAK/STAT pathway dysregulation in tendinopathy.

Mitochondrial dynamics imbalance has been identified as one of the key findings of this study. OPA1 was found to be downregulated, resulting in mitochondrial fragmentation and mtDNA leakage. The leaked mtDNA was shown to activate the cGAS/STING signaling, forming a vicious cycle of “inflammation–mitochondrial damage.” This mechanism has been previously reported in rheumatoid arthritis and neurodegenerative diseases; however, its role in the context of tendinopathy has been elucidated for the first time in this study.

Although the NF-κB signaling pathway is a well-established regulator of inflammation in various pathological contexts, including tendinopathy, our study focused primarily on the JAK2/STAT3-OPA1 axis due to its prominent inhibition observed in our GSEA analysis (**Figure 2F**) and experimental data. We acknowledge that NF-κB may also participate in tendinopathy progression, and it is possible that crosstalk exists between the JAK/STAT and NF-κB pathways. The potential interactions between these pathways will be explored in future, to provide a more comprehensive understanding of inflammatory regulation in tendinopathy.

From a therapeutic perspective, targeting PTP1B or its downstream pathways exhibits considerable potential. Animal experiments demonstrated that macrophage-specific knockout of Ptpn1 significantly alleviated tendon inflammation and fibrosis, while also improving tissue mechanical properties. These results are consistent with the therapeutic effects of PTP1B inhibitors in metabolic disorders, suggesting that both local and systemic inhibition of PTP1B may be a novel interventional avenue for tendinopathy. However, the potential side effects of PTP1B inhibitors are also worth noting. Studies have shown that PTP1B inhibition can enhance the insulin signaling pathway (32), which theoretically may lead to excessive insulin sensitivity and cause hypoglycemia. PTP1B is also expressed in the brain and is involved in leptin signaling, regulating appetite and energy metabolism and neurodevelopment (33). Inhibiting PTP1B may affect the function of the central nervous system, and potential side effects may include mood changes, cognitive impacts, etc. In addition, common side effects during the drug development process, including liver and kidney toxicity, gastrointestinal reactions, and allergic reactions, are also important factors restricting the clinical use of PTP1B inhibitors.

Several limitations of this study should be acknowledged. First, although bioinformatics analysis revealed the synergistic roles of M1 macrophages and T cells, the specific mechanisms underlying their mutual regulation remain undefined. Recent studies have shown that obesity induced by a high-fat diet can exacerbate collagenase-induced tendon injury and increase the levels of IL-1β and MMPs (34), highlighting that the abnormal energy metabolism and immune inflammation may be important contributing factors to tendinopathy. In addition, the precise role of immune effectors downstream of the cGAS/STING pathway in tendinopathy requires further investigation. Finally, the function of PTP1B in tenocytes or other stromal cells has not been independently studied, and potential multicellular crosstalk may influence the design of therapeutic strategies.

In conclusion, this study has systematically elucidated the molecular mechanism by which macrophage PTP1B regulates mitochondrial dynamics via the JAK2/STAT3-OPA1 axis and activates the cGAS/STING signaling pathway, thereby offering a novel perspective on the pathological progression of tendinopathy. These findings contribute to offer new insights in the mechanisms underlying tendinopathy, and provide a theoretical basis for developing precision therapies targeting PTP1B or its associated pathways. Future research should further investigate multicellular interactions, metabolic reprogramming, and immune activation, and promote translational studies to assess the clinical relevance of these findings.

## Data Availability

Publicly available datasets were analyzed in this study. This data can be found here: https://www.ncbi.nlm.nih.gov/geo/query/acc.cgi?acc=GSE26051, https://ngdc.cncb.ac.cn/search/specific?db=hra&q=HRA002325.

## References

[1] Dean BJF Dakin SG Millar NL Carr AJ. Review: Emerging concepts in the pathogenesis of tendinopathy. Surgeon. (2017) 15:349–54. doi: 10.1016/j.surge.2017.05.005, PMID: 28619548 PMC5714045

[2] Han Q Bai L Qian Y Zhang X Wang J Zhou J. Antioxidant and anti-inflammatory injectable hydrogel microspheres for in *situ* treatment of tendinopathy. Regenerative Biomaterials. (2024) 11:rbae007. doi: 10.1093/rb/rbae007, PMID: 38414798 PMC10898336

[3] Umamahesvaran B Sambandam SN Mounasamy V Gokulakrishnan PP Ashraf M. Calcifying tendinitis of shoulder: A concise review. J orthopaedics. (2018) 15:776–82. doi: 10.1016/j.jor.2018.05.040, PMID: 29946204 PMC6014564

[4] Rees JD Stride M Scott A. Tendons - time to revisit inflammation. Br J Sports Med. (2014) 48:1553–U54. doi: 10.1136/bjsports-2012-091957, PMID: 23476034 PMC4215290

[5] Sunwoo JY Eliasberg CD Carballo CB Rodeo SA. The role of the macrophage in tendinopathy and tendon healing. J Orthopaedic Res. (2020) 38:1666–75. doi: 10.1002/jor.24667, PMID: 32190920

[6] Zhang W Fang X Liu Y Liu C Yao C Guo J. Sulforaphane modulates macrophage polarization via JAK1/STAT1 inhibition to promote tendon repair in tendinopathy. Int Immunopharmacol. (2025) 163:115302. doi: 10.1016/j.intimp.2025.115302, PMID: 40763479

[7] Li DM Li S He SK He HP Yuan GX Ma BB. Restoring tendon microenvironment in tendinopathy: Macrophage modulation and tendon regeneration with injectable tendon hydrogel and tendon-derived stem cells exosomes. Bioactive Materials. (2025) . 47:152–69. doi: 10.1016/j.bioactmat.2025.01.016, PMID: 39906648 PMC11791013

[8] Millar NL Hueber AJ Reilly JH Xu YH Fazzi UG Murrell GAC. Inflammation is present in early human tendinopathy. Am J Sports Med. (2010) 38:2085–91. doi: 10.1177/0363546510372613, PMID: 20595553

[9] Feng HT He ZY Twomey K Ilaltdinov AW Leong D Wang YH. Epigallocatechin-3-gallate suppresses pain-related and proinflammatory mediators in the subacromial bursa in rotator cuff tendinopathy. Discov Med. (2019) 27:63–77., PMID: 30825883

[10] Xu JX Zheng MZ Feng ZX Lin QJ. CCL4L2 participates in tendinopathy progression by promoting macrophage inflammatory responses: a single-cell analysis. J Orthopaedic Surg Res. (2024) 19:836. doi: 10.1186/s13018-024-05268-9, PMID: 39696421 PMC11656782

[11] Cheng LX Zheng QQ Qiu KJ Ker DFE Chen X Yin Z. Mitochondrial destabilization in tendinopathy and potential therapeutic strategies. J Orthopaedic Translation. (2024) 49:49–61. doi: 10.1016/j.jot.2024.09.003, PMID: 39430132 PMC11488423

[12] Liao SY Chen L Song ZY He H. The fate of damaged mitochondrial DNA in the cell. Biochim Et Biophys Acta-Molecular Cell Res. (2022) 1869:119233. doi: 10.1016/j.bbamcr.2022.119233, PMID: 35131372

[13] Jiang LL Liang JC Wang TH Meng FF Duan WM. ETS proto-oncogene 1 modulates PTP1B expression to participate in high glucose-mediated endothelial inflammation. Acta Biochim Et Biophys Sin. (2022) 54:565–73. doi: 10.3724/abbs.2022021, PMID: 35607953 PMC9827757

[14] Yang L Sun YY Liu YR Yin NN Bu FT Yu HX. PTP1B promotes macrophage activation by regulating the NF-κB pathway in alcoholic liver injury. Toxicol Lett. (2020) 319:11–21. doi: 10.1016/j.toxlet.2019.11.001, PMID: 31711802

[15] van Montfort RLM Congreve M Tisi D Carr R Jhoti H. Oxidation state of the active-site cysteine in protein tyrosine phosphatase 1B. Nature. (2003) 423:773–7. doi: 10.1038/nature01681, PMID: 12802339

[16] Wang AH Ma HY Yi YL Zhu SJ Yu ZW Zhu J. Oleanolic acid derivative alleviates cardiac fibrosis through inhibiting PTP1B activity and regulating AMPK/TGF-β/Smads pathway. Eur J Pharmacol. (2023) 960:176116. doi: 10.1016/j.ejphar.2023.176116, PMID: 38059443

[17] Xiong H Du W Zhang YJ Hong J Su WY Tang JT. Trichostatin A, a histone deacetylase inhibitor, suppresses JAK2/STAT3 signaling via inducing the promoter-associated histone acetylation of SOCS1 and SOCS3 in human colorectal cancer cells. Mol Carcinogenesis. (2012) 51:174–84. doi: 10.1002/mc.20777, PMID: 21520296

[18] Beales ILP Ogunwobi OO. Glycine-extended gastrin inhibits apoptosis in Barrett’s oesophageal and oesophageal adenocarcinoma cells through JAK2/STAT3 activation. J Mol Endocrinol. (2009) 42:305–18. doi: 10.1677/jme-08-0096, PMID: 19153190

[19] Deng X Ye F Zeng LX Luo WZ Tu S Wang XY. Dexmedetomidine Mitigates Myocardial Ischemia/Reperfusion-Induced Mitochondrial Apoptosis through Targeting lncRNA HCP5. Am J Chin Med. (2022) 50:1529–51. doi: 10.1142/s0192415x22500641, PMID: 35931662

[20] Supasai S Adamo AM Mathieu P Marino RC Hellmers AC Cremonini E. Gestational zinc deficiency impairs brain astrogliogenesis in rats through multistep alterations of the JAK/STAT3 signaling pathway. Redox Biol. (2021) 44:102017. doi: 10.1016/j.redox.2021.102017, PMID: 34049221 PMC8167189

[21] Tsunekawa T Banno R Mizoguchi A Sugiyama M Tominaga T Onoue T. Deficiency of PTP1B attenuates hypothalamic inflammation via activation of the JAK2-STAT3 pathway in microglia. Ebiomedicine. (2017) 16:172–83. doi: 10.1016/j.ebiom.2017.01.007, PMID: 28094236 PMC5474442

[22] Bedalov A Salvatori R Dodig M Kronenberg MS Kapural B Bogdanovic Z. Regulation of COL1A1 expression in type I collagen producing tissues: identification of a 49 base pair region which is required for transgene expression in bone of transgenic mice. J Bone mineral Res. (1995) 10:1443–51. doi: 10.1002/jbmr.5650101004, PMID: 8686499

[23] Islam A Mbimba T Younesi M Akkus O. Effects of substrate stiffness on the tenoinduction of human mesenchymal stem cells. Acta Biomaterialia. (2017) 58:244–53. doi: 10.1016/j.actbio.2017.05.058, PMID: 28602855 PMC5551443

[24] Guo YZ You YH Shang FF Wang XW Huang B Zhao BY. iNOS aggravates pressure overload-induced cardiac dysfunction via activation of the cytosolic-mtDNA-mediated cGAS-STING pathway. Theranostics. (2023) 13:4229–46. doi: 10.7150/thno.84049, PMID: 37554263 PMC10405855

[25] Scaini G Mason BL Diaz AP Jha MK Soares JC Trivedi MH. Dysregulation of mitochondrial dynamics, mitophagy and apoptosis in major depressive disorder: Does inflammation play a role? Mol Psychiatry. (2022) 27:1095–102. doi: 10.1038/s41380-021-01312-w, PMID: 34650203

[26] Sánchez-Rodríguez R Tezze C Agnellini AHR Angioni R Venegas FC Cioccarelli C. OPA1 drives macrophage metabolism and functional commitment via p65 signaling. Cell Death Differentiation. (2023) 30:742–52. doi: 10.1038/s41418-022-01076-y, PMID: 36307526 PMC9984365

[27] Chen L Liu TT Tran A Lu XY Tomilov AA Davies V. OPA1 mutation and late-onset cardiomyopathy: mitochondrial dysfunction and mtDNA instability. J Am Heart Assoc. (2012) 1:e003012. doi: 10.1161/jaha.112.003012, PMID: 23316298 PMC3541627

[28] Liu CY Han YH Gu XM Li M Du YY Feng N. Paeonol promotes Opa1-mediated mitochondrial fusion via activating the CK2α-Stat3 pathway in diabetic cardiomyopathy. Redox Biol. (2021) 46:102098. doi: 10.1016/j.redox.2021.102098, PMID: 34418601 PMC8385203

[29] Fu F Liu CY Shi R Li M Zhang M Du YY. Punicalagin protects against diabetic cardiomyopathy by promoting opa1-mediated mitochondrial fusion *via* regulating PTP1B-stat3 pathway. Antioxidants Redox Signaling. (2021) 35:618–41. doi: 10.1089/ars.2020.8248, PMID: 33906428

[30] Ploeger C Huth T Sugiyanto RN Pusch S Goeppert B Singer S. Prohibitin, STAT3 and SH2D4A physically and functionally interact in tumor cell mitochondria. Cell Death Dis. (2020) 11:1023. doi: 10.1038/s41419-020-03220-3, PMID: 33257655 PMC7705682

[31] Mohammed F Gorla M Bisoyi V Tammineni P Sepuri NBV. Rotenone-induced reactive oxygen species signal the recruitment of STAT3 to mitochondria. FEBS Lett. (2020) 594:1403–12. doi: 10.1002/1873-3468.13741, PMID: 31981230

[32] Feng B Zhang J Liu Z Xu Y Hu HB. Discovery and biological evaluation of novel dual PTP1B and ACP1 inhibitors for the treatment of insulin resistance. Bioorganic Medicinal Chem. (2024) 97:117545. doi: 10.1016/j.bmc.2023.117545, PMID: 38070352

[33] Ge X Hu MM Zhou ML Fang XL Chen X Geng DQ. Overexpression of forebrain PTP1B leads to synaptic and cognitive impairments in obesity. Brain Behav Immun. (2024) . 117:456–70. doi: 10.1016/j.bbi.2024.02.008, PMID: 38336024

[34] Ling SKK Liang ZR Lui PPY. High-fat diet-induced obesity exacerbated collagenase-induced tendon injury with upregulation of interleukin-1beta and matrix metalloproteinase-1. Connective Tissue Res. (2024) . 65:447–57. doi: 10.1080/03008207.2024.2409751, PMID: 39364694

