# Supplementary material for: Macrophage PTP1B regulates mitochondrial dynamics via the JAK2/STAT3-OPA1 axis and activates the cGAS/STING signaling pathway
Source: Front Immunol. 2025 Oct 8;16:1644289. doi: 10.3389/fimmu.2025.1644289 (PMC12540072; doi:10.3389/fimmu.2025.1644289)
Supplement: Supplementary file 1 [file DataSheet1.docx]

**Supplementary information**

**Macrophage PTP1B Regulates Mitochondrial Dynamics via the JAK2/STAT3-OPA1 Axis and Activates the cGAS/STING Signaling Pathway**


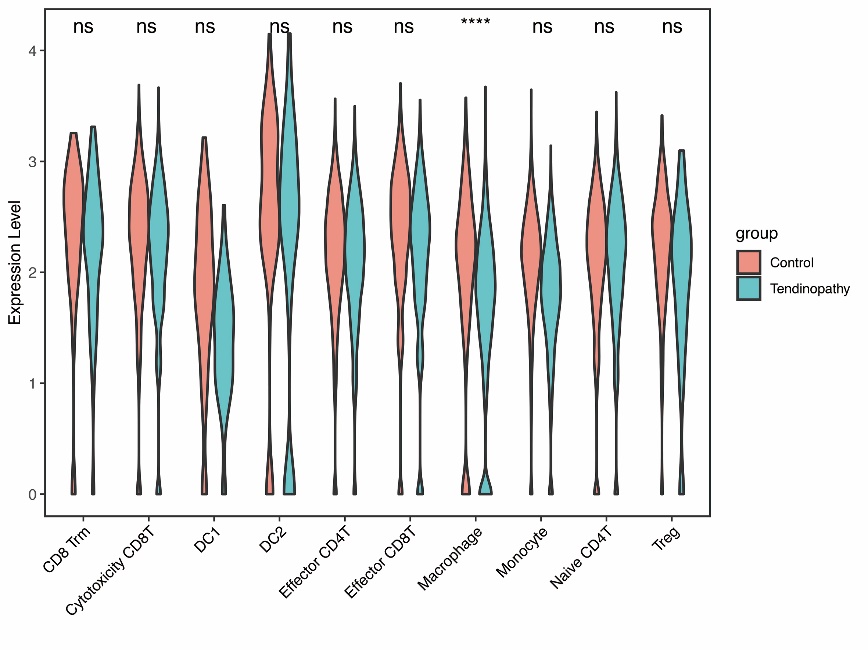


Figure S1 Expression levels of Ptpn1 genes of the 10 cell types we identified in Control and Tendinopathy.


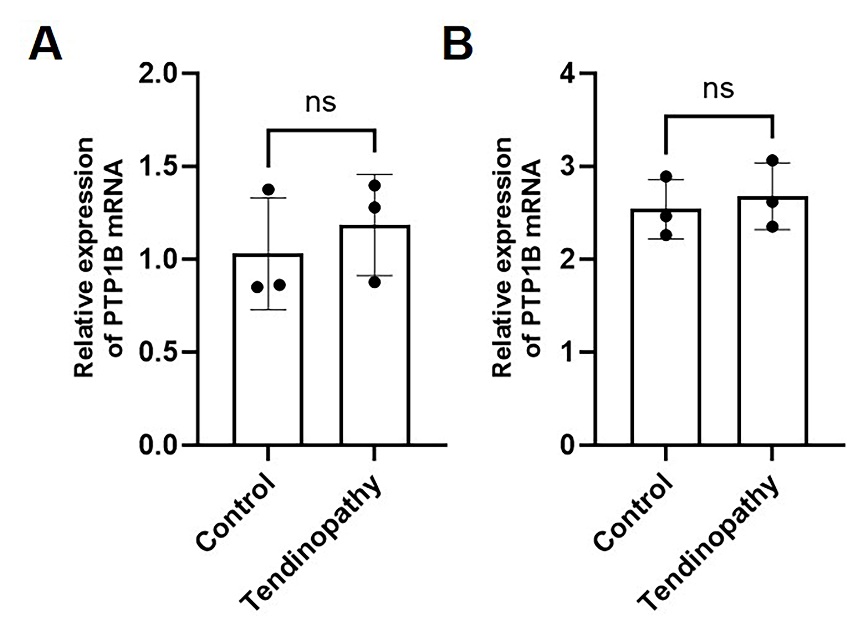


Figure S2 The mRNA expression level of PTP1B in (A) synovium and (B) skeletal muscles (n = 3).


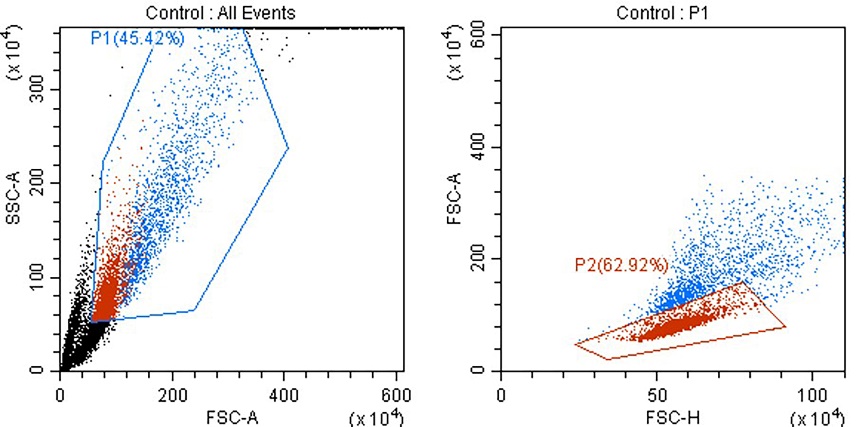


Figure S3 Gating strategies for flow cytometry in Figure 3I.
