# Supplementary material for: Macrophage PTP1B regulates mitochondrial dynamics via the JAK2/STAT3-OPA1 axis and activates the cGAS/STING signaling pathway
Source: Front Immunol. 2025 Oct 8;16:1644289. doi: 10.3389/fimmu.2025.1644289 (PMC12540072; doi:10.3389/fimmu.2025.1644289)
Supplement: Supplementary file 2 [file DataSheet2.pdf]

frontiers

The International Society for the Advancement of Cytometry (ISAC) has highlighted the importance of including comprehensive methodological information to ensure data reproducibility and reliability. In line with this, Frontiers in Immunology now requires authors to submit a checklist for manuscripts that involve flow or mass cytometry. This checklist helps standardize the reporting process, improving the quality and transparency of published data. By doing so, we support scientific progress, making it easier for other researchers to replicate and validate experiments.

This form should be submitted with any manuscripts using flow or mass cytometry.

Sample/specimen/material description

☐ Total blood

☐ PBMCs

☐ Organ digests

Other Tendon tissue from tendinopathy mice

Did the samples suffer any treatment before or after incubation with the antibodies?

☐ Drug None

☐ Cell permeabilization None

☐ Dye None

☐ Propidium iodine

☑ Not applicable

Other None

Instrument and antibodies

Name of the Cytometer BD FACSAria III

Antibodies and targets	Fluorochrome/ Metal	Catalog number/Company
e.g. anti-CD4	FITC	Cat. XXX/ XXX ltd.
anti-CD86	Alexa Fluor@594	ab303578/abcam

frontiers

anti-CD206	Alexa Fluor@594	ab318808/abcam
anti-F4/80	FITC	ab105155/abcam

Data analyses

1. Name of the software FlowJo v10___________________
2. Reference gating strategy in the manuscript or supplementary material

Gating strategy in (eg Figure X) _Reference gating strategy in the manuscript
